# Exploring the Relationship between Fibromyalgia-Related Fatigue, Physical Activity, and Quality of Life

**DOI:** 10.3390/ijerph19084870

**Published:** 2022-04-17

**Authors:** Marcos C. Alvarez, Maria Luiza L. Albuquerque, Henrique P. Neiva, Luis Cid, Filipe Rodrigues, Diogo S. Teixeira, Rui Matos, Raúl Antunes, Verónica Morales-Sánchez, Diogo Monteiro

**Affiliations:** 1 Department of Sport Sciences, University of Beira Interior, 6201-001 Covilhã, Portugal; marcos.alvarez@ubi.pt (M.C.A.); mluiza.laurentino@gmail.com (M.L.L.A.); hpn@ubi.pt (H.P.N.); 2 Research Center in Sport, Health and Human Development (CIDESD), Trás os Montes and Alto Douro University, 5000-558 Vila Real, Portugal; luiscid@esdrm.ipsantarem.pt; 3 Sport Sciences School of Rio Maior, Polytechnic of Santarém (ESDRM-IPSantarém), 2040-413 Rio Maior, Portugal; 4 Life Quality Research Centre (CIEQV), 2400-901 Leiria, Portugal; filipe.rodrigues@ipleiria.pt (F.R.); rui.matos@ipleiria.pt (R.M.); raul.antunes@ipleiria.pt (R.A.); 5 ESECS—Polytechnic of Leiria, 2411-901 Leiria, Portugal; 6 Faculty of Physical Education and Sport, Lusófona University (ULHT/FEFD), 1749-024 Lisbon, Portugal; diogo.teixeira@ulusofona.pt; 7 Research Center in Sport, Physical Education, and Exercise and Health (CIDEFES), 1749-024 Lisbon, Portugal; 8 Center for Innovative Care and Health Technology (ciTechCare), 2415-396 Leiria, Portugal; 9 Department of Social Psychology, Social Work, Social Anthropology and East Asian Studies, Faculty of Psychology, Malaga University, 29003 Malaga, Spain; vomorales@uma.es

**Keywords:** fibromyalgia, quality of life, fatigue, physical activity

## Abstract

The symptoms of fibromyalgia are varied, including general muscle pain and pain at specific points (also called tender points), excessive fatigue, anxiety, depression, and some psychological problems that can have a negative impact on quality of life. Physical activity is a widely used option by health professionals to alleviate the effects of this syndrome. However, there is no clear information on the possible mediating role of physical activity in the relationship between fibromyalgia-related fatigue and quality of life. Therefore, this study aims to evaluate the relationship between fibromyalgia-related fatigue and quality of life, and to investigate the mediating role of physical activity in patients with this syndrome. Methods: In a cross-sectional study, 237 Portuguese women aged 28 to 75 years (M = 49.12; SD ± 8.87) and 117 Brazilian women aged 20 to 69 years (M = 46.72; SD ± 8.38) were recruited to participate in this study. These patients completed three valid and reliable questionnaires related to the assessment of fibromyalgia-related fatigue (MDF-Fibro-17), physical activity (IPAQ), and quality of life (SF-36). Results: Both samples had scores above the midpoint for all dimensions of fibromyalgia-related fatigue and scores below the midpoint for quality of life. Physical activity had no mediating effect in either sample, as the total indirect effect was not significant. Conclusions: Physical activity does not mediate the relationship between fatigue and quality of life. However, the results also show that the fatigue dimensions associated with fibromyalgia had a negative and significant association with physical and mental health indicators in both samples. Thus, patients with FM with higher scores on fatigue-related symptoms might suffer more from physical and mental health, both of which are related to quality of life.

## 1. Introduction

Fibromyalgia Syndrome (FM) is defined as a neurological, physical, and chronic condition that causes sensory changes and muscle pain [1]. However, its pathogenesis is still debated by experts. Hyperexcitability of the central nervous system and neurotransmitter imbalance is seen as the main causes [2,3]. FM affects between 2% and 6% of the world’s population, with middle-aged women (between 30 and 50 years old) seeming to be the most affected [4,5]. One of the main characteristics of FM is its symptomatologic diversity namely: generalized muscle pain in specific points (called tender points); excessive fatigue (which does not improve even with prolonged rest); reduced muscle strength [6,7]; some psychological adversities: sleep problems, anxiety, depression, low self-esteem, and lower levels of satisfaction with life [8,9]. The studies developed by Gracely et al. [10] and Jensen et al. [11] verified, through imaging exams, that FM patients have a different brain function compared to healthy individuals. These FM patients have a higher activation and mild brain distortion in areas of pain control [10,11,12]. Furthermore, the study conducted by Lutz et al. [13] demonstrated that patients with FM have less gray matter in brain areas that regulate pain signals.

Due to this complexity, many patients have difficulties in describing their complaints and symptoms and, sometimes, health professionals do not identify the patient’s chronic pain as fibromyalgia-related pain or still do not believe in this condition reported by patients [12]. By itself, the diagnosis is complex, due to a lack of consistent assessment tools and means to accurately diagnose FM [14]. To obtain this diagnosis, the patient must reach certain values in two questionnaires, one focused on the severity of symptoms, the Symptom Severity Scale (SSS), and another focused on measuring the levels of generalized muscle pain, the Expanded Pain Index (WPI) [13]. After combining the criteria obtained in these questionnaires and without symptomatic changes for at least three months, the patient is diagnosed with FM [15]. The studies developed by Clauw [9] and White et al. [16] found that establishing the diagnosis of fibromyalgia in the early stages can help health professionals to create effective interventions to reduce fibromyalgia-related symptoms, decrease health care costs. In addition, effective diagnosis can reduce tests to search for the causes of the pain reported by these patients.

### 1.1. Fatigue and Quality of Life

Fatigue is widely known and understood, as it is a natural response of the body to some type of physical and mental stress, but it can also be a sign of some possible physical and/or mental disorder [17,18]. In healthy individuals, fatigue is a physiological reaction to a prolonged activity, from which the body tends to recover easily with rest, and which usually does not end up interfering with daily activities. However, in individuals affected by some pathology (e.g., FM, anemia, hypothyroidism, chronic obstructive pulmonary disease) or with physical limitations, fatigue symptoms are more pronounced when compared to healthy individuals [17]. Specifically, patients with FM report that their fatigue is characterized by excessive physical, mental, and cognitive tiredness and that it is usually not alleviated after hours of sleep or rest, which may end up hindering the performance of work or daily tasks, and thus may contribute to the adoption of sedentary behavior [17,19,20].

Several studies (e.g., Arnold et al. [19]; Hudson et al. [21]) have shown that fatigue is one of the main symptoms reported by patients with FM when asked about the determining factor impacting their overall health, quality of life, and overall perception of this syndrome. While fibromyalgia-related fatigue has a negative relationship with several health-related indicators such as quality of life, it is not always measured in clinical practice and scientific research [22,23].

Because of its complexity, FM leaves patients more vulnerable to stigmatization and psychological conditions (e.g., poor quality of life, higher levels of anxiety and depression, and low self-esteem) as evidenced by Garcia-Martinez et al. [24]. Patients with FM have significantly worse levels of quality of life when compared to healthy individuals [24]. These patients see their syndrome as a serious condition that entails major consequences for their daily lives, affecting their socialization, making it difficult for them to work and perform their daily tasks [25].

### 1.2. The Mediation Role of Physical Activity

There is still no cure or fully effective treatment for FM, and the most recommended intervention for this type of population is the implementation of approaches that can reduce fibromyalgia-related symptoms [8,26]. In this sense, physical activity has been pointed out by health professionals as a means to alleviate the effects of this syndrome [8,26]. Physical activity is recommended since it is considered a low-cost intervention, aimed at improving physical and psychological components, through the increase of some neurotransmitters (i.e., endorphins and serotonin) capable of producing a sense of self-efficacy and satisfaction, promoting thus a better quality of life [27,28].

Health professionals are advised to consider the physical limitations of each patient and measure the levels of physical fitness and fibromyalgia-related fatigue as a means to understand possible pain caused by the training program [28,29]. Therefore, research recommends a frequency of 2–3 times a week of low- to moderate-intensity physical activity for patients with FM [28,29]. Some studies have shown that aerobic and strength exercises have moderate effects on physical functioning, can reduce fibromyalgia-related pain (by increasing muscle strength) and increase flexibility, as well as improve quality of life [6,30].

### 1.3. Present Study

As previously described, FM is a chronic and complex biopsychosocial disorder that deteriorates and compromises the quality of life, and several studies [31,32,33] demonstrate that those patients may have these lower levels, which are influenced by several psychological aspects, such as depression and fatigue. Some studies [34,35] demonstrated that patients with FM tend to have worse health status and quality of life when compared to other patients with other chronic diseases (e.g., osteoarthritis, rheumatoid arthritis, lupus, hypertension, spondylarthritis, and Sjogren’s Syndrome). Studies [36,37] found that women with FM, when compared to healthy women, present significant differences in quality of life values, especially in the domains of functional capacity, vitality, emotional role, pain, and general health perception.

However, to the best of our knowledge, the association between fibromyalgia-related fatigue and quality of life is still under-researched. As demonstrated by Offenbaecher et al. [38], more than 50% of FM patients reported that their quality of life levels are not related to pain, but to other aspects of FM.

Daily physical activity is an important behavioral mechanism that can influence fatigue directly or indirectly, through changes in neuroendocrine activities (increased levels of serotonin and other neurotransmitters, regulation of the hypothalamic-pituitary-adrenal axis, and the autonomic nervous system) and, consequently, it can help improve quality of life levels due to its role in promoting well-being, increasing levels of general health status, and improving some psychological aspects in cases where there is social interaction during physical exercise [39,40]. However, when the literature on the mediating role of physical activity in this relationship between fatigue and quality of life was consulted, a scarcity of specific literature was observed. Therefore, this study aims to evaluate the relationship between fatigue and quality of life in patients, as well as investigate how physical activity can act as a mediator of this relationship.

## 2. Materials and Methods

### 2.1. Study Design and Participants

A cross-sectional study considering two independent samples was collected for the present study. Sample 1 consisted of a total of 237 Portuguese women aged between 28 and 75 (*M* = 49.12; *SD* ± 8.87) years was conducted. The Portuguese subjects were diagnosed with FM on average 8.62 ± 7.14 years ago. Sample 2 consisted of data from 117 Brazilian women aged between 20 and 69 years (*M* = 46.72; *SD* = ± 8.38). The Brazilian subjects were diagnosed with FM on average 8.91 ± 6.92 years ago. According to Fritz and Mackinnon [41], the present sample size is in line with simulations for mediation purposes with this number of variables, thus, ensuring proper statistical power.

### 2.2. Procedure: Data Collection

Prior to data collection, ethical approval was obtained from the Ethics and Scientific Committee of the University of Beira Interior (UBI), under reference number CE-UBI-Pj-2021-038. The present study was conducted in accordance with the Declaration of Helsinki [42] and its subsequent amendments.

Regarding data collection procedures, the National Association against Fibromyalgia and Chronic Fatigue Syndrome (MYOS) and the Brazilian Association of Fibromyalgia (ABRAFIBRO) were contacted, and the objectives were explained. After approval, FM specialists contacted potential participants to participate voluntarily in this study. Afterward, the authors contacted patients with FM and explained the purpose of this study, along with providing detailed information regarding data collection procedures. After contact and clarification of the study objectives, informed consent was obtained from each individual participant. All individuals voluntarily participated in this study, and the time to complete the questionnaire was approximately 25 min.

### 2.3. Instruments

#### 2.3.1. Fibromyalgia-Related Fatigue

The Multidimensional Daily Diary of Fatigue-Fibromyalgia-17 MDF-Fibro-17 Portuguese and Brazilian versions [43] were used to measure the levels of different fatigue components in patients with FM. The 17 items assess different dimensions of fatigue: Global Fatigue Experience (4 items, for example, “How severe is your fatigue today?”); Physical Fatigue (3 items, for example, “How weak your muscles felt today?”); Cognitive Fatigue (4 items, e.g., “How hard was it to concentrate because you were tired today?”); Motivation (3 items, for example, “How much effort was it doing today?”), and Role Impact (3 items, for example, “Did you slow things down because you were tired today?”). Participants responded to each item using a 10-point scale ranging from 0 (“not at all”) to 10 (“extremely”). Higher scores indicated greater severity of fatigue. Previous studies have supported the validity and reliability of these questionnaires [43,44].

#### 2.3.2. Physical Activity

The International Physical Activity Questionnaire short form (IPAQ) was used to assess the levels of physical activity [45]. The questionnaire comprises a total of seven questions, related to activities carried out in the last seven days before the application of the questionnaire [46].

The questions measure principles of physical activity, such as walking, moderate-intensity and vigorous-intensity activities, frequency, and duration [46,47]. The estimation of energy expenditure is processed according to the levels of physical activity [48]. Specifically, the data obtained are converted to MET min/week (i.e., metabolic equivalent task) by calculating the scored minutes per week in each activity category by the specific metabolic equivalent, according to previous research [46].

#### 2.3.3. Quality of Life

The SF-36 survey [49] was used to measure the quality of life dimensions. This survey consists of 11 questions and 36 items that encompass 8 components (domains or dimensions) on the quality of life state, represented by: physical functioning (10 items, for example, “Does your health limit you to performing violent activities, such as running, lifting weights, participating in strenuous sports? If yes, how much?”); Difficulties in Role Performance Caused by Physical Problems (4 items, for example, “During the past 4 weeks have you, in your work or daily activities, had decreased time spent working or in other activities as a result of your physical health status?”); Pain (2 items, for example, “During the past 4 weeks, how much has pain interfered with your normal work? (both outside work and housework)”); General Health (5 items, for example, “In general, I would say your health is”); Vitality (4 items, for example, “During the last 4 weeks, have you felt full of vitality?”); Social Functioning (2 items, for example, “During the past four weeks, how much has your physical health or emotional problems limited your social activity? (such as visiting friends or close family)”); Difficulties in Role Performance Caused by Emotional Problems (3 items, for example, “During the past 4 weeks, have you had, with your work or daily activities, a decrease in time spent at work or in other activities due to any emotional problems? (such as feeling depressed or anxious)”); Mental Health (5 items, for example, “During the past 4 weeks, have you felt so depressed that nothing cheered you up?”) [50]. To verify the physical health status of the patient, a calculation is made of the following domains: Physical Functioning, Difficulties in Role Performance Caused by Physical Problems, Pain, and General Health. As for the psychological health status, the calculation is made from the domains: Vitality, Social Functioning, Difficulties in Role Performance Caused by Emotional Problems, and Mental Health. Values range from 0 to 100, where higher scores suggest better levels in each domain of the patient’s quality of life, and mental and physical health [51].

### 2.4. Statistical Analysis

Descriptive statistics (mean and standard deviation), as well as bivariate correlations, were calculated for all variables under analysis. The IBM SPSS Statistics version 22.0 (IBM Corp. Armonk, New York, NY, USA) was used for data analysis. Possible missing values and outliers were also searched in the data.

To test the proposed interactions, simple mediation (model 4) analysis according to Hayes’ [52] recommendations was conducted using SPSS PROCESS v.3.5 (IBM Corp. Armonk, New York, NY, USA). In total, twenty models were tested, considering five fatigue dimensions as independent variables and two dependent variables in two independent samples. Specifically, the levels of Global Fatigue Experience (GFE); Physical Fatigue (PF); Motivation (MOT), and Impact on Function (IF) were selected as independent variables in this model. The levels of physical activity (PA) were used as a mediator, and the levels of Physical Health (PH) and Mental Health (MH) were selected as dependent variables. This procedure allows the estimation of the direct and indirect effects in the proposed models while controlling for k mediators’ influence between variables [52]. A 5000 samples bootstrap was used according to several recommendations [52,53] and significant indirect effects were considered if the confidence interval did not include zero. For all tests, the level of significance was set at *p* < 0.05.

## 3. Results

### 3.1. Descriptive Statistics and Bivariate Correlations

Data inspection did not show missing values and outliers. Descriptive statistics showed that both samples had scores above the midpoint for the experience of Global Fatigue, Physical Fatigue, Cognitive Fatigue, Motivation, and Impact on Function. The observed values of fibromyalgia-related fatigue dimensions are similar in both samples as can be seen in Table 1. Furthermore, the Portuguese sample seems to be physically more active than the Brazilian patients (MET = 2573.54 vs. MET = 1734.15). In terms of quality of life (physical and mental health), both the Portuguese and Brazilian patients showed scores below average. Fibromyalgia-related fatigue dimensions displayed a negative and significant association with physical and mental health indicators.

### 3.2. The Mediation Role of Physical Activity in the Relationship between Fibromyalgia-Related Fatigue and Quality of Life

Fibromyalgia-related fatigue dimensions displayed a negative and significant association with physical and mental health indicators in both samples, since direct regression coefficients were negative and significant (*p* < 0.05). However, fibromyalgia-related fatigue dimensions did not display any significant association with levels of physical activity. The levels of physical activity did not display any significant association with physical and mental health in both samples. Overall, no mediation effect of physical activity was identified, since the total indirect effect was not significant in both samples (see Table 2). 

## 4. Discussion

The present study aimed to evaluate the influence of fatigue on the quality of life of patients with FM, and to verify whether the level of physical activity can mediate the association between fatigue and quality of life. First, the results of this study showed that women with FM scored above the midpoint on all components of fatigue in both samples. This has been observed in previous studies that showed that patients with FM have higher levels of fatigue and its components, especially at the muscular, cognitive, and emotional levels [20,54,55]. The studies conducted by Humphrey [20] and Öncü [56] have shown that fatigue at FM affects patients not only at the physical level, but also at the cognitive and psychosocial levels. These patients reported reduced mental resilience and slow thinking, which directly affects their quality of life [56].

Other studies show that patients with FM tend to be more aware of fatigue and its components compared to other pathologies [38,57,58,59]. For example, the study by Humphrey et al. [20] showed that patients described their fatigue as excessive, affecting their motivation to perform desired activities and even interfering with their ability to concentrate, think clearly, and remember certain things or themselves. This was confirmed by the patients in the study by Offenbaecher et al. [38]. They reported that fatigue is an important symptom of the disease, and that it has several important consequences that affect their functioning and well-being [38].

In terms of quality of life (physical and mental health), the results of the present study indicated that, both in the Brazilian and Portuguese sample, the scores were below the midpoint. This finding is supported by the existing literature [24,51,59], which demonstrates that patients with FM have a low perception of their quality of life. Garcia-Martinez et al. [24] found in their study that patients with FM had significantly worse levels of quality of life when compared to healthy people, which is understandable given the number of symptoms that patients with FM face. In addition, a study conducted by Wolfe et al. [59] verified that patients with FM have lower levels of quality of life when compared to patients with other systemic disorders (namely chronic obstructive pulmonary disease and insulin-dependent diabetes mellitus). Concomitantly, the study carried out by da Costa et al. [51] verified that women with FM had a lower perception of their quality life values when compared to women with systemic lupus erythematosus.

The studies conducted by Tander et al. [60] and Walker et al. [61] compared patients with FM and rheumatoid arthritis and found that patients with FM had higher values in fatigue and social isolation, and lower values in sleep quality and all parameters of the SF-36 in terms of quality of life. The study by Borman et al. [62] also found that patients with FM had lower levels of quality of life when compared to patients with rheumatoid arthritis. Another important finding was that patients with FM had lower levels of perceived ability to cope with their disease when compared with patients with rheumatoid arthritis. The study by Burckhardt et al. [63] corroborates these findings, as their study compared the quality of life among patients with FM, rheumatoid arthritis, and osteoarthritis, and found that patients with FM had much lower values in all domains of quality of life.

Neumann et al. [64] carried out a study comparing the quality of life between patients with FM, patients with generalized chronic pain syndrome, and healthy people, and demonstrated that patients with FM had the lowest values of quality of life. Picavet et al. [65] compared 12 different pathologies of the musculoskeletal system (i.e., herniated disc, gout, repetitive strain injury, epicondylitis, knee osteoarthritis, hip osteoarthritis, osteoporosis, whiplash, rheumatoid arthritis, fibromyalgia, tendonitis, and capsulitis), and found that all patients had low scores of quality of life. However, patients with rheumatoid arthritis and FM had the lowest values compared to other pathologies under analysis [65].

Yilmaz et al. [66] carried out a comparative study among four diseases (i.e., knee osteoarthritis, shoulder impingement syndrome, fibromyalgia, and osteoporosis), and found that patients with FM had low values in all quality of life scores. The study by Ataoğlu et al. [67] compared the quality of life of patients with FM, knee osteoarthritis, and rheumatoid arthritis, and found that patients with FM had lower values in all quality of life parameters compared to other diseases [66].

### 4.1. The Mediation Role of Physical Activity

In our study, it was possible to verify that physical activity does not play a mediating role in the relationship between fibromyalgia-related fatigue and quality of life. The study developed by Doerr et al. [39] corroborates our findings, where no association was found between the mediating role of physical activity concerning fatigue and other symptoms associated with FM. However, Sawatzy et al. [27] verified in their study that physical activity partially mediates the relationship between chronic diseases and quality of life. Another study with contradictory findings compared to ours was by Molinari et al. [68] who found that physical activity acted as a possible mediator between FM symptoms and patients’ quality of life. These results demonstrated the need for further studies to verify if physical activity can act as a mediator between fibromyalgia-related fatigue and quality of life in patients with FM [68].

Indeed, physical activity is considered an effective tool of controlling the symptoms of FM. It could reduce fatigue, pain, depression, and anxiety, and could promote improvement in quality of life [6,69,70,71].

However, research still differs on what intensity of physical activity is best for FM patients. For instance, the studies conducted by Gavilán-Carrera et al. [72] and Buman et al. [73] have shown that patients with FM have better control of fibromyalgia-related symptoms when they engage in low-intensity physical activity. Further, the studies developed by Fanning et al. [74] and Kaleth et al. [75] demonstrated that patients with FM showed improvement in quality of life and relieved symptoms of the syndrome when they participate in a high-intensity physical activity program. However, Clauw [9] argued that moderate-intensity physical activity is the most effective treatment for the management of fibromyalgia-related symptoms. Therefore, Cunha Ribeiro et al. [76] suggested that exercise programs should be individualized. That is, the intensity should be considered based on the patient’s limitations, level of physical fitness, and fatigue. In this regard, Rooks [29] found that the health benefits of physical activity in patients with FM are increased and/or maintained compared to those patients who are physically inactive. Some studies [28,29] have shown that FM patients undergoing group exercises improved their physical, emotional, and social capacities. Therefore, it is reasonable to assume that the physical activity intensity could act as a possible mediator, however, the type of intensity of physical activity should be further investigated in the future.

Despite all the benefits of physical activity for this type of population, training programs must be supervised by a qualified professional [77]. For example, King et al. [78] and Ayan et al. [79] found that patients with FM who adhered to the supervised physical exercise protocol showed an improvement in their ability to manage the FM symptoms. In addition, Sevimli et al. [80] found that patients who exercise under supervision had a significant reduction in pain and FM impact when compared to other patients who performed physical activity without supervision. One of the factors that may explain the difference between our results and the existing literature [77], is that the patients had difficulties in remembering and performing exercise programs without supervision, thus generating a fear that these exercises performed incorrectly could increase pain levels. The study by Kayo et al. [81] found that after the end of the supervised exercise program, there was low adherence to physical activity by patients, leading to a detraining effect. Thus, it seems that, for physical activity to influence FM symptoms and other related health indicators (e.g., quality of life), exercise programs should be supervised. Hence, we suspect that in our study, physical activity did not mediate the relationship between FM fatigue and both mental and physical health since physical activity was subjectively measured and not determined as supervised exercise.

For instance, the study by Carus et al. [82] demonstrated that in 3 months of exercise, patients demonstrate great relief from pain, emotional problems, an improvement in quality of life, physical functional capacity, and balance. This same study found that 12 weeks after the end of the experimental intervention, there was a decrease in physical fitness, as well as in some components of quality of life. This can demonstrate that the monitoring of physical activity can help to improve the quality of life and attenuate the symptoms of patients with FM.

The study by Salvat et al. [83] showed that patients who underwent a multidisciplinary treatment, involving supervised physical activity, showed an improvement in their functional status, and an increase in the level of physical activity. However, the study by Ramsay et al. [77] compared a supervised and unsupervised aerobic exercise program and found that the group that underwent the supervised training program had an improvement in measures of psychological well-being when compared to the other group. Another interesting finding of the study by Ramsay et al. [77] was that exercise adherence by the unsupervised group was very low when compared to the supervised exercise group.

The study conducted by Burckhardt et al. [84] compared the effects of an education program (where patients were instructed and performed the activity independently) with exercise monitoring and found that both groups showed improvements in quality of life and self-efficacy. However, only in the group that performed supervised physical activity was there also improvement in pain, fatigue, and muscle stiffness. King et al. [78] also suggested that for this type of population, it may be better to emphasize the adoption of a physically active lifestyle, with appropriate professional supervision, rather than just promoting exercise per se.

### 4.2. Practical Implications and Limitations

In recent years, scientific publications on FM have increased considerably, which can be explained by a greater awareness and shared interest from various stakeholders, including patients with FM, specific organizations for these groups, researchers, and professionals in the field of health and physical activity. Although our results show that it does not have a connection between the quality of life and fatigue, some studies [28,29,82] show that physical activity is an important tool in reducing fatigue levels and improving quality of life levels in patients with FM.

However, the physical activity prescribed for this population should be supervised and performed in an observational manner. Thus, the professional can adjust and adapt the intensity and volume, so that the patient can better adapt to this type of activity and prevent poor performance, avoiding any worsening of FM symptoms resulting from incorrect or exaggerated physical activity.

Firstly, this study had a cross-sectional design. Therefore, longitudinal/experimental studies may be useful in measuring fatigue symptoms and quality of life in FM patients. Another limitation of this study was the specificity of the sample, where only Portuguese and Brazilian patients took part in the research, meaning that the data obtained cannot be generalized and compared to other countries and cultures. In addition, the physical activity was evaluated through a self-reported way (i.e., questionnaire). It means that no definition of intensity was considered, nor whether there was any type of professional supervising the activity. Therefore, future studies should try to address this limitation to confirm the importance of professional supervision in performing physical activities for these groups.

The age group presented between patients in both samples should be considered as another limitation, since some studies [85,86] suggest that patients with younger ages tend to have higher levels of fatigue and lower levels of quality of life when compared to older patients. As a suggestion for future research, studies could be carried out on how age can influence the quality of life, fatigue, and levels of physical activity in patients with FM.

Finally, one more limitation is the participation of male patients, since in the current study, only female participants were included. This decision was made considering the existing literature which demonstrates that FM has a greater impact in women compared with men, so we opted for a more conservative approach, solely with female patients with FM [87,88]. Nevertheless, future studies should try to collect some data on male FM patients to analyze these associations between gender and explore their impact across cultures.

## 5. Conclusions

Overall, the results suggest that physical activity does not mediate the association between quality of life and fatigue in patients with FM. However, the results also show that fatigue dimensions associated with fibromyalgia have negative and significant associations with physical and mental health indicators in both samples. Thus, FM patients with higher scores on fatigue-related symptoms might suffer more from physical and mental health, both of which are related to quality of life.

## Figures and Tables

**Table 1:** Descriptive statistics and bivariate correlations across study variables.

Portuguese Sample										
Variables	M	SD	1	2	3	4	5	6	7	8
1.GFE	7.93	1.52	1	-	-	-	-	-	-	-
2.PF	8.13	1.52	0.81 **	1	-	-	-	-	-	-
3.CF	7.78	1.71	0.66 **	0.67 **	1	-	-	-	-	-
4.MOT	8.13	1.54	0.75 **	0.75 **	0.77 **	1	-	-	-	-
5.IF	8.20	1.52	0.73 **	0.74 **	0.76 **	0.86 **	1	-	-	-
6.PA	2573.54	675.04	−0.05	−0.003	−0.04	0.03	0.020	1	-	-
7.MH	32.78	9.31	−0.24 **	−0.27 **	−0.31 **	−0.32 **	−0.28 **	0.03	1	-
8.MH	27.41	15.48	−0.15 **	−0.14 *	−0.30 **	−0.33 **	−0.29 **	−0.01	0.50 **	-
Brazilian sample										
Variables	M	SD	1	2	3	4	5	6	7	8
1.GFE	8.06	1.44	1	-	-	-	-	-	-	-
2.PF	8.33	1.46	0.68 **	1	-	-	-	-	-	-
3.CF	7.95	1.50	0.66 **	0.51 **	1	-	-	-	-	-
4.MOT	8.49	1.48	0.60 **	0.54 **	0.76 **	1	-	-	-	-
5.IF	8.42	1.59	0.65 **	0.59 **	0.70 **	0.83 **	1	-	-	-
6.PA	1734.15	368.12	0.03	−0.05	−0.10	−0.06	−0.05	1	-	-
7.MH	33.81	8.85	−0.16 **	−0.12 *	−0.17 *	−0.17 *	−0.14 *	−0.13 *	1	-
8.MH	26.90	15.83	−0.023 **	−0.21 **	−0.22 **	−0.16 *	−0.16 *	−0.18 *	0.23 **	-

Notes: M = Mean; SD = Standard deviation; GFE = Global Fatigue Experience; PF = Physical Fatigue; CF = Cognitive Fatigue; MOT = Motivation; IF = Impact on Function; PH = Physical Health; MH = Mental Health; PA = Physical Activity; * *p* < 0.05; ** *p* < 0.01.

**Table 2:** Mediation analysis between fibromyalgia-related fatigue, physical activity, and quality of life.

Portuguese Sample						
Path 1	Effect	Path 2	Effect	Path 3	Effect	Total Indirect Effect
GFE→PH	−0.17 (−0.251, −0.078)	GFE→PA	0.03 (−0.057, 0.120)	PA→PH	0.04 (−0.088, 0.161)	0.01 (−0.04, 0.010)
GFE→MH	−0.11 (−0.191, −0.015)	GFE→PA	0.03 (−0.057, 0.120)	PA→MH	−0.06 (−0.133, 0.120)	−0.01 (−0.06, 0.04)
PF→PH	−0.18 (−0.255, −0.093)	PF→PA	−0.01 (−0.086, 0.823)	PA→PH	0.02 (−0.098, 0.148)	<0.01 (−0.07, 0.05)
PF→MH	−0.09 (−0.174, −0.07)	PF→PA	−0.01 (−0.086, 0.823)	PA→MH	−0.01 (−0.140, 0.113)	<0.01 (−0.05, 0.03)
CF→PH	−0.18 (−0.253, −0.110)	CF→PA	−0.02 (−0.097, 0.052)	PA→PH	0.01 (−0.108, 0.135)	−0.01 (−0.016, 0.028)
CF→MH	−0.17 (−0.245, −0.102)	CF→PA	−0.02 (−0.097, 0.052)	PA→MH	−0.02 (−0.146, 0.098)	0.01 (−0.017, 0.010)
MOT→PH	−0.20 (−0.283, −0.126)	MOT→PA	0.02 (−0.062, 0.102)	PA→PH	0.04 (−0.086, 0.157)	0.01 (−0.05, 0.010)
MOT→MH	−0.22 (−0.704, −0.329)	MOT→PA	0.02 (−0.062, 0.102)	PA→MH	−0.03 (−0.123, 0.118)	−0.01 (−0.06, 0.03)
IF→PH	−0.18 (−0.263, −0.101)	IF→PA	0.01 (−0.070, 0.097)	PA→PH	0.03 (−0.091, 0.154)	<0.01 (−0.04, 0.08)
IF→MH	−0.19 (−0.270, −0.109)	MOT→PA	0.02 (−0.062, 0.102)	PA→MH	−0.07 (−0.129, 0.115)	−0.01 (−0.05, 0.02)
Brazilian sample						
Path 1	Effect	Path 2	Effect	Path 3	Effect	Total indirect effect
GFE→PH	−0.19 (−0.259, −0.103)	GFE→PA	0.02 (−0.088, 0.129)	PA→PH	0.03 (−0.128, 0.188)	0.01 (−0.03, 0.011)
GFE→MH	−0.16 (−0.266, −0.053)	GFE→PA	0.02 (−0.088, 0.129)	PA→MH	0.05 (−0.109, 0.198)	0.01 (−0.04, 0.012)
PF→PH	−0.17 (−0.245, −0.112)	PF→PA	−0.03 (−0.140, 0.075)	PA→PH	0.03 (−0.127, 0.189)	−0.01 (−0.018, 0.03)
PF→MH	−0.14 (−0.243, −0.030)	PF→PA	−0.03 (−0.140, 0.075)	PA→MH	0.03 (−0.127, 0.183)	−0.01 (−0.022, 0.03)
CF→PH	−0.15 (−0.241, −0.098)	CF→PA	−0.07 (−0.172, 0.036)	PA→PH	0.05 (−0.054, 0.156)	−0.02 (−0.022, 0.07)
CF→MH	−0.14 (−0.244, −0.037)	CF→PA	−0.07 (−0.172, 0.036)	PA→MH	0.02 (−0.139, 0.171)	−0.01 (−0.022, 0.011)
MOT→PH	−0.08 (−0.167, −0.05)	MOT→PA	0.06 (−0.127, 0.248)	PA→PH	0.04 (−0.122, 0.194)	−0.01 (−0.014, 0.07)
MOT→MH	−0.11 (−0.210, −0.013)	MOT→PA	0.06 (−0.127, 0.248)	PA→MH	0.03 (−0.128, 0.185)	−0.01 (−0.057, 0.155)
IF→PH	−0.12 (−0.201, −0.023)	IF→PA	−0.04 (−0.135, 0.063)	PA→PH	0.03 (−0.129, 0.188)	−0.01 (−0.08, 0.07)
IF→MH	−0.10 (−0.190, −0.09)	IF→PA	−0.04 (−0.135, 0.063)	PA→MH	0.03 (−0.127, 0.186)	−0.01 (−0.010, 0.09)

Notes: Path 1 = Path between independent and dependent variables; Path 2 = Path between independent variable and mediator; Path 3 = Path between mediator and dependent variable; GFE = Global Fatigue Experience; PF = Physical Fatigue; CF = Cognitive Fatigue; MOT = Motivation; IF = Impact on Function; PH = Physical Health; MH = Mental Health; PA = Physical Activity. For exploratory purpose and since results were similar between samples, the authors examined the model with both samples (total sample). However, the results did not differ significantly from those reported in the manuscript. Thus, we maintained current results from each sample for clarity.

## Data Availability

Due to issues of participant consent, data will not be shared publicly. Interested researchers may contact the corresponding author (diogo.monteiro@ipleiria.pt).
